# A Rare Case of Primary Adenocarcinoma of the Gallbladder With Lymphoid Stroma

**DOI:** 10.7759/cureus.46448

**Published:** 2023-10-03

**Authors:** Kaushik Saha, Dhiraj Kanti Das, Niladri Sarkar

**Affiliations:** 1 Department of Pathology, Murshidabad Medical College and Hospital, Berhampore, IND; 2 Department of General Surgery, Murshidabad Medical College and Hospital, Berhampore, IND

**Keywords:** gallbladder adenocarcinoma, lymphoid stroma, adenocarcinoma, stroma, lymphoid, gallbladder

## Abstract

Adenocarcinoma of the gallbladder is the most common gallbladder carcinoma. But lymphoid stroma in gallbladder carcinoma is one of the rarest presentations. A unique case of gallbladder adenocarcinoma with lymphoid stroma in a 47-year-old female is presented in this report. The surgically resected gallbladder demonstrated invasive adenocarcinoma with lymphoid stroma, though it was radiologically diagnosed as xanthogranulomatous cholecystitis. Adenocarcinoma was immunohistochemically positive for pancytokeratin (AE1/AE3), cytokeratin 7 (CK7), cytokeratin 20 (CK20), and carcinoembryonic antigen (CEA). Lymphoid stroma was positive for CD45, where B-cell zones were CD20 and CD79a positive, and T-cell zones were CD3 positive, with a larger T-cell subset being positive for CD4 than CD8. This is the fourth reported case of gallbladder adenocarcinoma with lymphoid stroma, which needs to be studied for pathogenesis, prognosis, and future therapy, if any.

## Introduction

The most common malignancy of the biliary tract is carcinoma of the gallbladder. The most common type of gallbladder carcinoma is adenocarcinoma [1], but lymphoid stroma in gallbladder carcinoma is one of the rarest presentations. Prominent reactive lymphoid infiltration is a relatively common entity in nonkeratinizing undifferentiated nasopharyngeal carcinoma previously known as lymphoepithelial carcinoma [2], invasive breast carcinoma of no special type (IBC-NST) with a medullary pattern recognized earlier as medullary carcinoma [3], and gastric adenocarcinoma with lymphoid stroma [4]. We report here a unique case of gallbladder adenocarcinoma with lymphoid stroma. This is probably the fourth reported case of gallbladder carcinoma with lymphoid stroma [5-8].

## Case presentation

**Figure 1:**
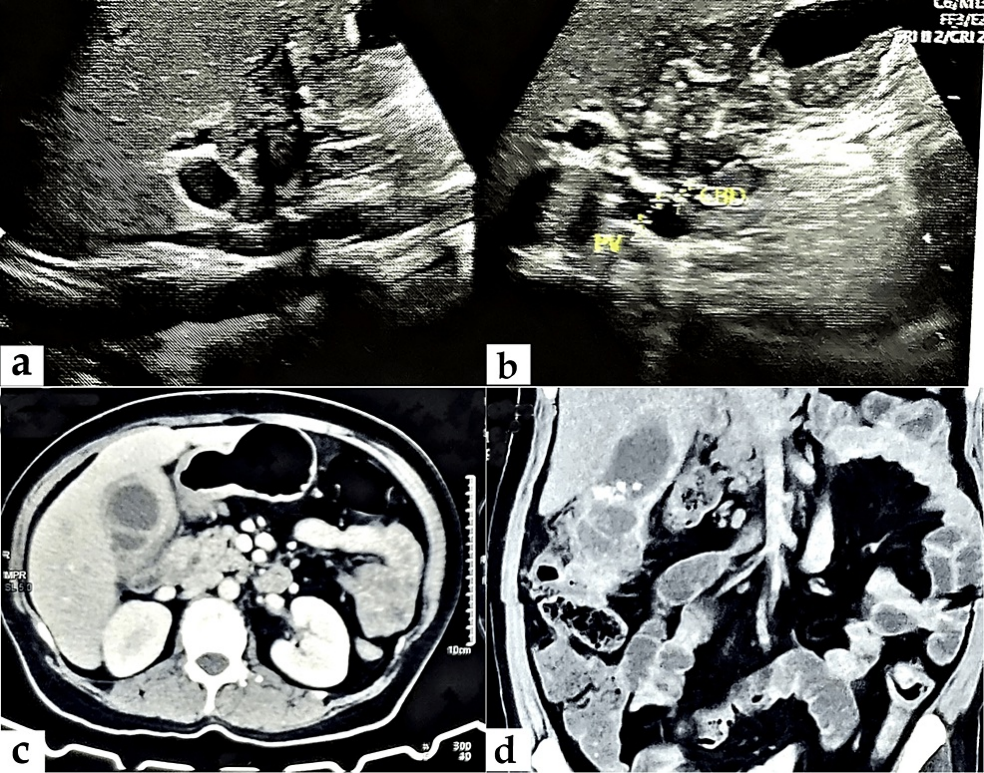
(a) and (b) Gray-scale US images show irregular thickening of the gallbladder wall; (c) axial view of the CECT image; and (d) coronal view of the CECT image showing multiple small calculi, abnormal gallbladder wall thickening, loss of a fat plane with the adjacent liver, and hypoattenuating intramural nodules. US: ultrasound; CECT: contrast-enhanced CT

A non-diabetic, normotensive 47-year-old female patient presented with recurrent pain in the upper abdomen and an occasional fever with chills and rigor for the last six months. Blood reports were normal except for mild leukocytosis. Upper abdominal ultrasonography (USG) revealed irregular thickening of the gallbladder wall, favoring xanthogranulomatous cholecystitis. A contrast-enhanced CT (CECT) scan demonstrated intraluminal multiple small calculi, abnormal gallbladder wall thickening, pericholecystic fat stranding, loss of a fat plane with the adjacent liver segment, and small hypoattenuating intramural nodules favoring xanthogranulomatous cholecystitis more than a neoplastic lesion (Figure 1).

**Figure 2:**
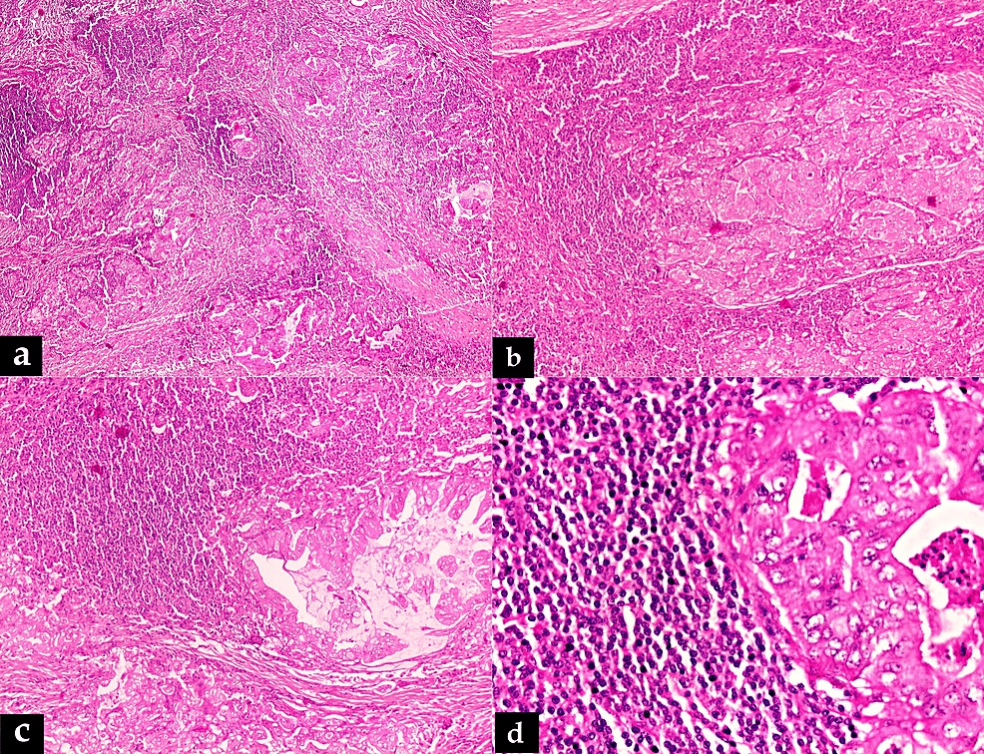
(a) Photomicrograph showing peri-tumoral lymphoid cuffing and thick fibrous bands in between the tumor islands (H and E, ×40); (b) and (c) low-power view of adenocarcinoma with lymphoid stroma (H and E, ×100); (d) high-power view of atypical malignant glands with benign-looking lymphoid stroma (H and E, ×400).

Finally, the patient underwent laparoscopic cholecystectomy, but the surgeon couldn’t dissect the entire gallbladder wall from the hepatic bed. A whitish, solid, homogenous mass was detected on gross examination, measuring approximately 5.0 × 4.5 × 2.0 cm. The case was diagnosed histologically as moderately differentiated adenocarcinoma involving the liver bed as well as the serosal aspect on the other side. High-grade biliary intraepithelial neoplasia (BilIN) was noted in part of the gallbladder lining epithelium. The conspicuous lymphoid stroma cuffing the invasive tumor islands was diffusely seen throughout the thickened gallbladder wall (Figure 2).

**Figure 3:**
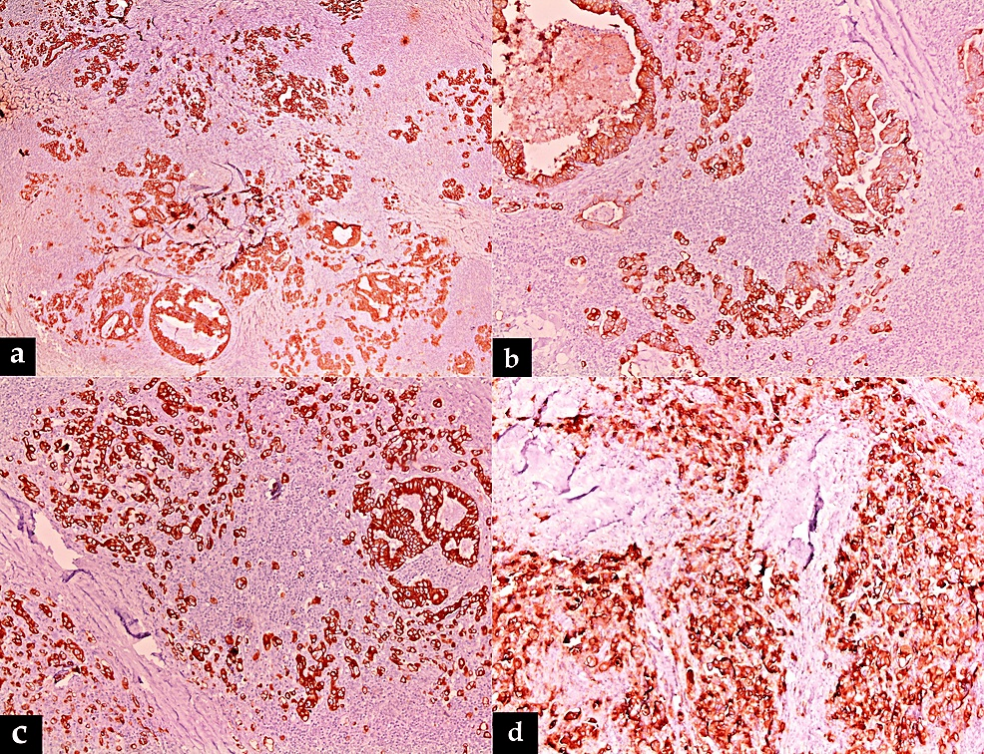
Positive immunostaining of adenocarcinoma with (a) pancytokeratin (×40), (b) CK7 (×100), (c) CK20 (×100), and (d) CEA (×100) CK7: cytokeratin 7; CK20: cytokeratin 20; CEA: carcinoembryonic antigen

Thick fibrous bands were found in between the invasive tumor islands. The lymphoid stroma appeared to be reactive in nature without any atypical lymphoid cells or Reed-Sternberg-like cells. The adenocarcinoma component was diffusely positive for pancytokeratin (AE1/AE3), cytokeratin 7 (CK7), cytokeratin 20 (CK20), and carcinoembryonic antigen (CEA) (Figure 3).

**Figure 4:**
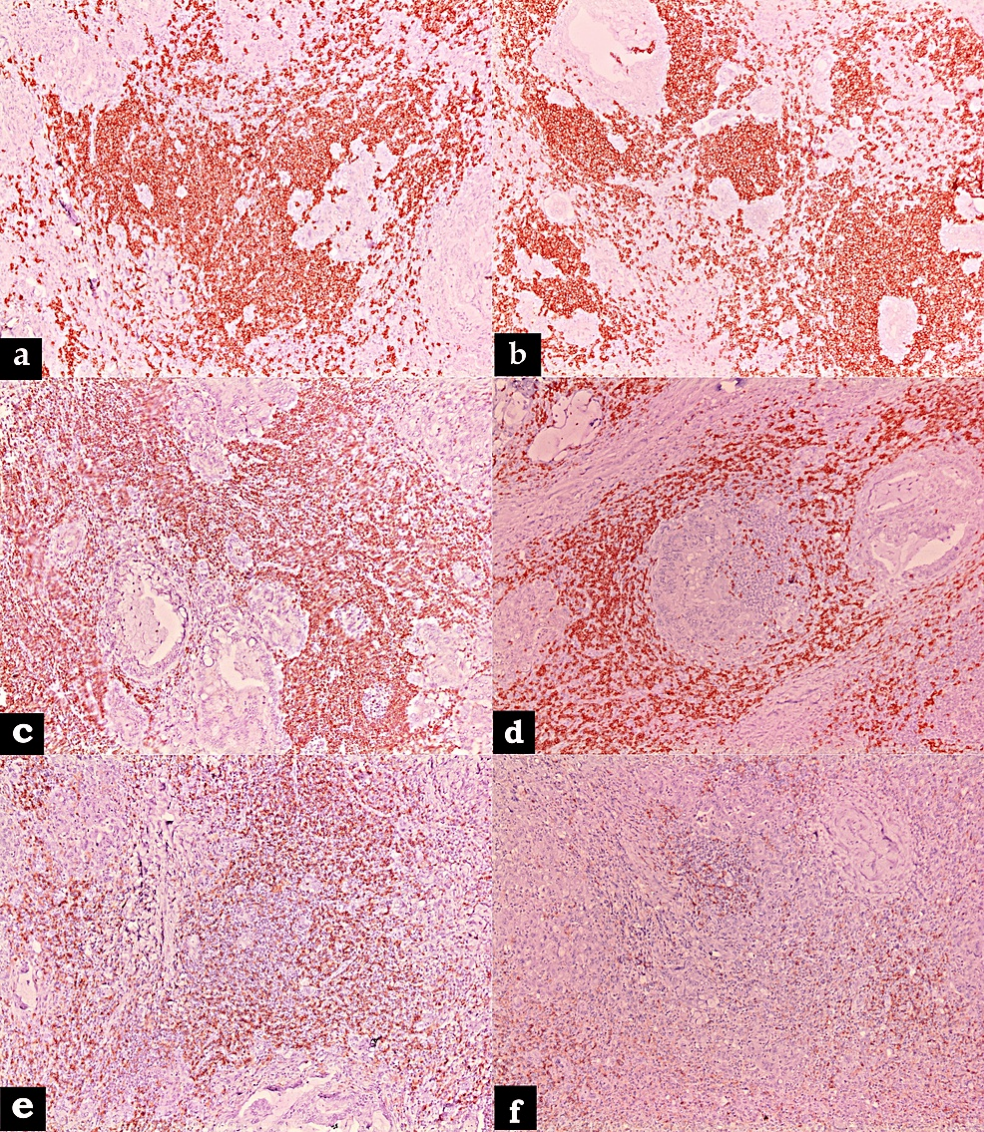
Immunostaining of lymphoid stroma with (a) CD45 (×100), (b) CD20 (×100), (c) CD79a (×100), (d) CD3 (×100), (e) CD4 (×100), and (f) CD8 (×100).

Lymphoid stroma was diffusely positive for CD45. Pan B-cell markers like CD20 and CD79a were positive in B-cell predominant areas admixed with pan T-cell marker CD3 positive cells, and a few scattered positives for CD138 positive plasma cells were also noted. Relatively more T-cell subsets displayed CD4 immunoreactivity than CD8 (Figure 4).

## Discussion

Gallbladder adenocarcinoma is a relatively common malignancy in our institute. But we first incidentally noticed the reactive lymphoid stroma throughout the thickened gallbladder wall, embracing all the invasive tumor islands. We retrieved three such reported cases from Japan. Muto et al. [5] reported the first case; Kijima et al. [6] observed the second case; and Sakai et al. [7] found the third case, which was also associated with spindle cell morule-like features. This is the first case outside of Japan.

Tumor-associated stromal lymphocyte infiltration can be explained by the interaction between carcinoma cells and host lymphocytes, where cytokines play an important pivotal role [5-7]. Several studies have been conducted in gastric and nasopharyngeal carcinomas to establish the prognosis, molecular pathology, and the association of Epstein-Barr virus (EBV) with lymphoid stroma [6]. A significant association has been found between EBV infection and gastric and nasopharyngeal carcinomas with lymphoid stroma [2,4]. However, no such study was carried out, probably due to the rarity of this finding in the gallbladder.

## Conclusions

This is a very unique finding in gallbladder carcinoma and the first overall case outside of Japan. The lymphoid stroma may have a definite relationship with the prognosis of the associated malignancy, like other organs, and it may have an EBV association in pathogenesis. Hence, this rare feature of gallbladder carcinoma needs to be studied for pathogenesis, prognosis, demographic pattern, and future therapy, if any, in multiple centers worldwide, as the number of cases for a single-center study is small.
